# Clinical Outcomes of Image-Guided Spinal Laser Interstitial Thermal Therapy for Metastatic Epidural Spinal Disease: A Systematic Review and Narrative Synthesis

**DOI:** 10.7759/cureus.114604

**Published:** 2026-08-16

**Authors:** Shahtajarab M Saltanat, Mohammed S Millat, Mohammed F Millat

**Affiliations:** 1 Department of Orthopaedics, Broomfield Hospital, Chelmsford, GBR; 2 Department of Orthopaedics, Princess Royal University Hospital, Orpington, GBR; 3 Department of Trauma and Orthopaedics, Broomfield Hospital, Chelmsford, GBR

**Keywords:** image-guided therapy, malignant spinal cord compression, metastatic epidural spinal cord compression, minimally invasive spine oncology, spinal laser interstitial thermal therapy, spinal metastases

## Abstract

Metastatic epidural spinal cord compression (MESCC) causes pain, neurological deficits, and reduced quality of life. While open surgery and radiotherapy are standard treatments, frail patients are often poor surgical candidates. Image-guided spinal laser interstitial thermal therapy (sLITT) has emerged as a minimally invasive alternative to reduce epidural tumor burden and facilitate radiotherapy.

We systematically searched PubMed, Embase, Scopus, and Web of Science (inception to 31 January 2026) for clinical reports on image-guided sLITT for malignant epidural spinal disease treated with decompressive intent. Two reviewers extracted data, with a third resolving discrepancies. Meta-analysis was precluded by clinical heterogeneity and potential cohort overlap, yielding seven included studies.

The included studies evaluated standalone magnetic resonance-guided sLITT, hybrid sLITT with percutaneous stabilization, and sLITT combined with stereotactic radiotherapy. Reported outcomes suggested short-term improvements in epidural tumor thickness and epidural spinal cord compression (ESCC) grade, neurological stability, brief hospitalizations, and lower perioperative resource use compared to open surgery in one matched observational study. However, safety reporting was heterogeneous, and the certainty of evidence was very low due to small sample sizes, retrospective designs, short follow-up, and inconsistent outcome measurements.

Image-guided sLITT appears feasible for carefully selected patients with metastatic epidural disease, but evidence regarding durable neurological benefit, survival, and long-term local control remains uncertain. Larger, prospective multicenter studies utilizing standardized neurological, radiographic, safety, and patient-reported outcomes are required.

## Introduction and background

Malignant spinal cord compression (MSCC) and metastatic epidural spinal cord compression (MESCC) are clinically urgent complications of advanced cancer. They usually arise from the epidural extension of vertebral metastatic disease and may produce pain, sensory disturbance, weakness, loss of ambulation, and permanent neurological disability. Imaging-based diagnosis, early recognition, multidisciplinary management, and timely local treatment are central because neurological recovery is closely linked to baseline neurological status, degree of epidural compression, speed of intervention, and feasibility of durable local control [1-4].

Management is individualized. Corticosteroids, radiotherapy, open decompression, separation surgery, and stabilization remain important treatment options, depending on neurological status, tumor radiosensitivity, spinal instability, prognosis, systemic disease burden, and surgical fitness [2,3]. Open decompression and stabilization can provide effective neural decompression and mechanical support, but may be associated with blood loss, wound morbidity, longer recovery, and delayed systemic or radiation therapy in selected patients [5].

Minimally invasive spine oncology has developed to reduce procedural morbidity while maintaining local control and symptom palliation. Laser interstitial thermal therapy (LITT) has been reviewed as part of the broader development of minimally invasive thermal therapy for tumors of the brain and spine, while image-guided vertebroplasty, kyphoplasty, radiofrequency ablation, and other percutaneous techniques have expanded treatment options for patients who may not tolerate extensive surgery [6-8]. Spinal laser interstitial thermal therapy (sLITT) applies percutaneously delivered laser energy to induce controlled thermal injury within tumor tissue. When performed with magnetic resonance imaging (MRI) guidance and real-time thermometry, sLITT may permit targeted ablation near the epidural space while monitoring thermal spread near neural structures [9-11].

The rationale for sLITT in metastatic epidural disease is not limited to tumor ablation. In appropriately selected cases, the technique may reduce epidural tumor volume, improve or stabilize epidural spinal cord compression (ESCC) grade as defined by the validated MRI-based ESCC scale [12], create a separation margin for stereotactic radiotherapy, and shorten recovery time compared with open procedures [9-11,13-17]. However, the evidence base remains limited by small observational cohorts, heterogeneous reporting, and repeated reports from the same institutional program. A prior systematic review summarized sLITT in metastases [17]. This review searched the literature through 31 January 2026, explicitly evaluated potential cohort overlap, separated primary clinical evidence from background reviews, organized findings by intervention pathway, and focused on epidural or neural-element disease rather than all spinal metastases.

## Review

Methods

Study Design and Reporting Framework

This systematic review with narrative synthesis was conducted and reported in accordance with the Preferred Reporting Items for Systematic Reviews and Meta-Analyses (PRISMA) 2020 statement [18]. Quantitative meta-analysis was not performed. Prospective registration was not required when the review was initiated and was therefore not undertaken. Consequently, no publicly accessible prospective protocol is available. To support transparency, the eligibility criteria, outcomes, complete search strategies, study-selection process, appraisal methods, and synthesis approach are reported in detail. The absence of prospective registration is acknowledged as a limitation because the review methods cannot be compared with a publicly available prespecified protocol.

Eligibility Criteria

Eligible studies were prospective or retrospective cohort studies, comparative observational studies, or case series reporting clinical outcomes after image-guided spinal laser therapy for malignant epidural spinal disease. Eligible populations included adults with spinal metastases or malignant spinal disease with definite ESCC, clinically suspected MSCC or MESCC, radiographic spinal canal compromise, or near-canal tumor extension when the laser procedure was performed with decompressive or neural-protective intent. Studies describing only tumor proximity without epidural involvement were considered separately during extraction and were not combined numerically with definite ESCC or MESCC cohorts.

The intervention of interest was image-guided spinal laser therapy, primarily magnetic resonance-guided sLITT with thermometry. Related computed tomography (CT)-guided or comparable image-guided laser thermal ablation approaches were eligible only when directed at epidural, near-canal, or neural-element-adjacent disease. The eligible treatment pathways comprised standalone sLITT, hybrid sLITT with percutaneous stabilization or instrumentation, and sLITT combined with stereotactic radiosurgery, stereotactic body radiotherapy, or conventional external beam radiotherapy. Case reports, purely technical descriptions without clinical outcomes, preclinical studies, conference abstracts without sufficient outcome data, low-level non-ablative laser therapy, benign spinal conditions, non-laser ablation studies, and intracranial-only LITT studies were excluded.

Information Sources and Search Strategy

PubMed, Embase, Scopus, and Web of Science were searched from database inception to 31 January 2026, and the final searches were run on 31 January 2026. No date or language restrictions were applied. The database-specific strategies combined terms for spinal metastasis or malignant epidural disease, spinal cord compression or canal compromise, and laser interstitial thermal therapy or laser thermal ablation. Reference lists of eligible studies and relevant reviews were screened, and forward citation tracking was performed when feasible. Complete reproducible search strategies, database platforms, search dates, limits, and yields are provided in Appendix A.

Study Selection and Data Extraction

Search results were combined, and duplicate records were removed before screening. Two reviewers performed title and abstract screening, full-text assessment, and data extraction using a standardized form. The extracted data and final eligibility decisions were reviewed by all three reviewers. Disagreements were resolved by majority opinion. Extracted data included study design, institution, sample size, recruitment period when reported, treated spinal levels, intervention pathway, adjunct radiotherapy or stabilization, baseline ESCC or neural-element involvement, neurological status, pain or quality-of-life outcomes, perioperative outcomes, complications, reintervention, local control, survival, and follow-up duration.

When reports originated from the same institution or clinical program, potential overlap was assessed using institution, recruitment period, sample size, intervention description, and outcome focus. Because overlap could not be definitively excluded for several reports, cumulative patient totals were not interpreted as unique individuals.

Outcomes

The primary outcomes were neurological or functional stability or improvement and radiographic decompression or response. Neurological and functional outcomes included absence of neurological deterioration, preservation of ambulation, Karnofsky Performance Status (KPS) [19], and neurological outcomes as defined in the individual source studies [9-11,13-16]. Radiographic outcomes included improvement in ESCC grade using the established ESCC classification [12] or reduction in epidural tumor thickness. Secondary outcomes included pain measured with the visual analogue scale (VAS) [20], health-related quality of life measured with the EuroQol five-dimension questionnaire (EQ-5D) [21], length of stay, estimated blood loss, operative time, time to radiotherapy, time to systemic therapy, complications, neurological deterioration, reoperation or other reintervention, local control, and overall survival.

Risk of Bias and Certainty of Evidence

Risk of bias was assessed according to study design. The matched non-randomized comparative study was evaluated using the Risk Of Bias In Non-randomized Studies of Interventions (ROBINS-I) tool [22]. The assessment considered bias due to confounding, participant selection, intervention classification, deviations from intended interventions, missing data, outcome measurement, and selection of the reported result. Non-comparative clinical series were assessed using the Joanna Briggs Institute (JBI) Critical Appraisal Checklist for Case Series [23], which considers inclusion criteria, standardized and valid condition measurement, consecutive and complete inclusion, reporting of participant characteristics and clinical information, outcome and follow-up reporting, site information, and appropriateness of statistical analysis. Appraisal disagreements were resolved by reviewer consensus. No numerical quality score or arbitrary cut-off was applied.

The certainty of evidence for each outcome domain was evaluated using the Grading of Recommendations Assessment, Development, and Evaluation (GRADE) approach [24]. Certainty judgments considered risk of bias, inconsistency, indirectness, imprecision, and publication bias. Because no pooled estimates were calculated, judgments were based on the consistency, directness, methodological limitations, and precision of the narrative evidence. Certainty was categorized as high, moderate, low, or very low.

Data Synthesis

Narrative synthesis was used for all outcomes. Meta-analysis was not performed because of the small number of studies, heterogeneous interventions and outcome definitions, possible cohort overlap, mixed patient-level and procedure-level reporting, and insufficient independent extractable data for consistent pooling. Results were organized by clinical domain and, where possible, by intervention pathway, including standalone sLITT, hybrid sLITT with stabilization, and sLITT integrated with radiotherapy. Review articles were used only for background, citation tracking, and contextual interpretation and were not treated as primary outcome-contributing studies.

Results

Study Selection

**Figure 1:**
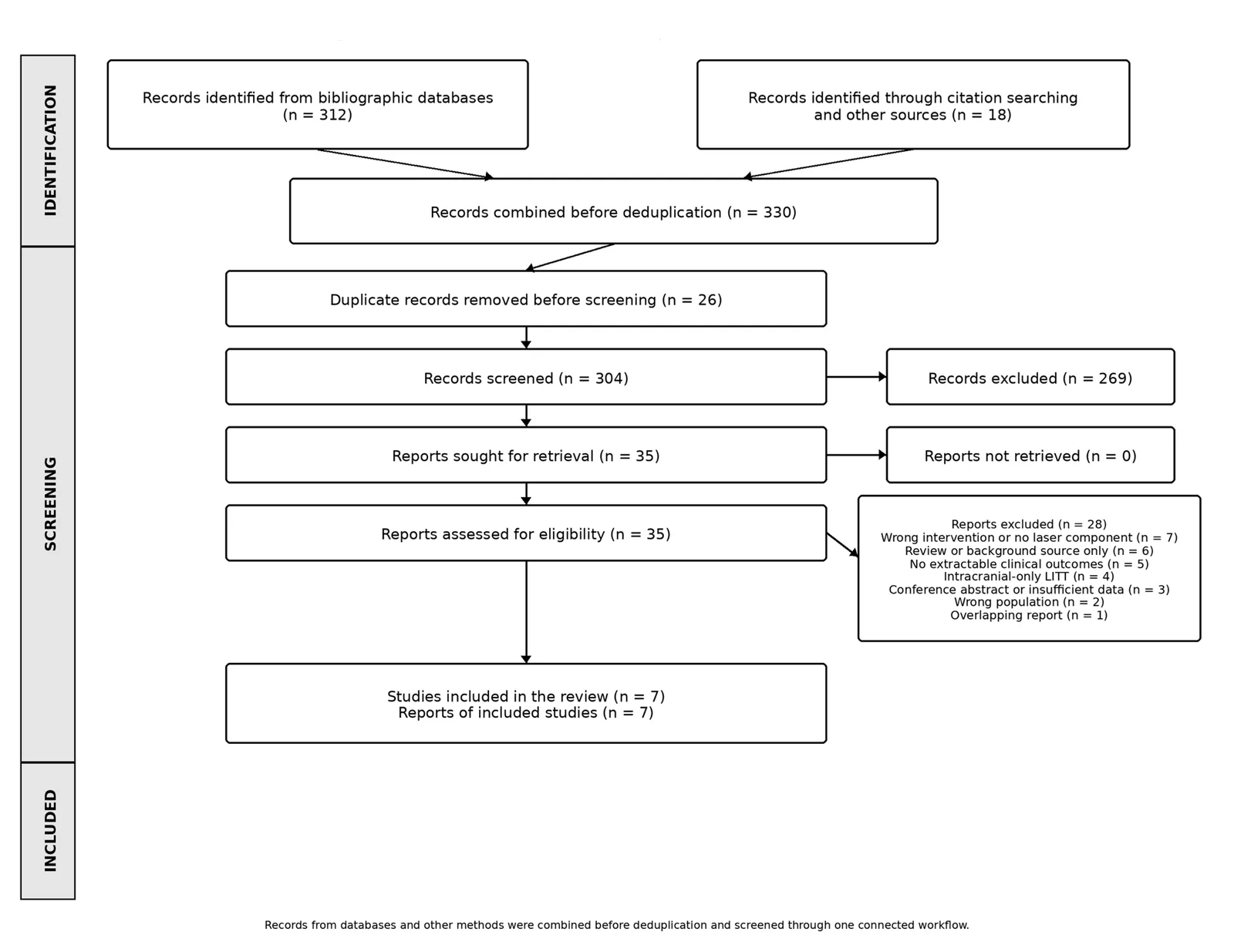
PRISMA 2020 flow diagram of the study selection PRISMA: Preferred Reporting Items for Systematic Reviews and Meta-Analyses; LITT: laser interstitial thermal therapy

The January 2026 search identified 312 records from bibliographic databases and 18 additional records through citation searching and other sources. These 330 records were combined before deduplication. After the removal of 26 duplicates, 304 records were screened, of which 269 were excluded. Thirty-five reports were sought and retrieved for full-text assessment; 28 were excluded for the reasons listed in Appendix B. Seven primary clinical reports were included in the narrative synthesis, and no study was included in the quantitative meta-analysis. The connected study-selection pathway is shown in Figure 1.

Records from databases and other methods were combined before deduplication and screened through a single connected workflow. The diagram follows the PRISMA 2020 study-selection structure [18].

Characteristics of the Included Clinical Reports

Seven primary clinical reports were included. The evidence base comprised early single-center feasibility series, a technical accuracy study with clinical outcomes, a hybrid percutaneous stabilization series, a matched observational comparison with open surgery, a consecutive 120-case institutional experience, and a 2024 retrospective cohort evaluating sLITT with radiotherapy for thoracic MESCC. All reports originated from the same or a closely related institutional program; therefore, patient totals were not summed across studies. Study characteristics, intervention pathways, and key clinical outcomes are summarized in Table 1, while detailed study-level outcome data are presented in Table 2.

**Table 1:** Summary of the included studies and reported clinical outcomes ESCC: epidural spinal cord compression; EQ-5D: EuroQol five-dimension questionnaire; KPS: Karnofsky Performance Status; MESCC: metastatic epidural spinal cord compression; MRI: magnetic resonance imaging; NR: not reported or not extractable from the review dataset; sLITT: spinal laser interstitial thermal therapy; VAS: visual analogue scale The ESCC classification, KPS, VAS, and EQ-5D are cited in the Methods section [12,19-21].

Study	Study design and institution	Recruitment period and sample size	Treated spinal levels and population	Intervention pathway	Adjunct radiotherapy or stabilization	Baseline ESCC or neural-element involvement and neurological status	Pain or quality-of-life outcomes	Radiographic and neurological outcomes	Perioperative outcomes	Complications and reintervention	Local control, survival, and follow-up duration
Tatsui et al. (2015) [9]	Prospective clinical case series; MD Anderson Cancer Center, USA	Period NR; 11 patients	Spinal metastases with high-grade epidural disease; levels NR	Percutaneous magnetic resonance-guided sLITT	Postoperative stereotactic radiosurgery in all patients; no open stabilization described	ESCC grades 1c-3; baseline neurological details variably reported	VAS and EQ-5D improved at 30 and 60 days	Median ESCC grade improved from 2 to 1b; epidural thickness decreased; no permanent neurological morbidity	Median length of stay 2 days	One transient sensory deficit with transient spinal cord T2-weighted signal; resolved; reintervention NR	Median follow-up 4.7 months; imaging assessment at approximately 2 months; durable local control NR
Tatsui et al. (2016) [10]	Clinical case series; MD Anderson Cancer Center, USA	Period NR; 19 patients	Metastatic epidural compression; levels NR	Magnetic resonance-guided sLITT	Postoperative stereotactic radiotherapy	Patients selected without motor deficit; formal ESCC distribution not fully extractable	Clinical improvement reported; validated values NR	Tumor size decreased in most patients; no permanent neurological injury reported	Short recovery described; exact operative time, blood loss, and stay NR	No permanent neurological injury; reintervention NR	Short-to-intermediate follow-up reported in source; exact local-control and survival values NR
Tatsui et al. (2016) [13]	Technical note with clinical case series; MD Anderson Cancer Center, USA	Period NR; 8 patients	ESCC with spinal instability; levels NR	sLITT plus percutaneous instrumentation	Percutaneous stabilization; radiotherapy variably timed	ESCC with mechanical instability; neurological status variably reported	NR	Mean modified ESCC score improved from 4.5 ± 0.27 to 2.75 ± 0.37; KPS and clinical outcomes reported	Median length of stay 5 days	Complication and reintervention data not consistently extractable	Median imaging follow-up 2.1 months; local control and survival NR
Tatsui et al. (2017) [11]	Technical accuracy study with clinical case series; MD Anderson Cancer Center, USA	Period NR; 13 patients	Metastatic epidural or near-canal disease; levels NR	MRI navigation-guided sLITT	Hybrid oncological care in some patients; stabilization and radiotherapy not uniform	Baseline ESCC reported; detailed neurological distribution NR	NR	Median ESCC grade improved from 2 to 1b; median targeting radial error 0.7 mm; KPS and clinical outcomes reported	Median length of stay 1 day	Safety data reported in source; reintervention NR	Median imaging follow-up 4.4 months; local control and survival NR
de Almeida Bastos et al. (2020) [14]	Matched non-randomized observational comparison; MD Anderson Cancer Center, USA	Period NR; 80 patients: 40 sLITT and 40 open surgery	Thoracic metastatic ESCC grade ≥1c	sLITT as a minimally invasive separation strategy versus open surgery	Open-surgery comparator; stereotactic radiosurgery usually followed both pathways	Thoracic ESCC ≥1c; standardized neurological outcome reporting limited	NR	ESCC reduction in 72.5% of sLITT and 90% of open-surgery patients; neurological scale not uniformly reported	Blood loss 117 ± 84 vs 1,331 ± 1,219 mL; stay 3.4 ± 4.3 vs 9.0 ± 5.9 days; time to radiosurgery 7.8 ± 11.8 vs 35.9 ± 32.7 days; time to systemic therapy 24.7 ± 21 vs 59 ± 80 days	Overall complications 5% vs 35%; source-defined major 2.5% vs 10%; source-defined minor 2.5% vs 25%; reintervention NR	Follow-up, local control, and survival not consistently extractable from the review dataset
Bastos et al. (2021) [15]	Retrospective consecutive institutional case series; MD Anderson Cancer Center, USA	2013-2019; first 120 sLITT cases	Metastatic tumors causing ESCC; treated levels NR in review dataset	Program-level sLITT	Radiotherapy and other oncological care as clinically indicated	ESCC from metastatic tumors; neurological status reported at program level	NR	Program-level decompression and clinical outcomes reported	Approximately 85% discharged home within 48 hours	Overall complication rate 18%, mostly transient or procedural; reintervention reported in source but exact value NR	Program-level local-control outcomes reported; longer follow-up than early series; probable overlap with other reports
Tom et al. (2024) [16]	Retrospective cohort; MD Anderson Cancer Center, USA	2013-2022; 129 patients and 144 vertebral segments	Thoracic MESCC	sLITT integrated with radiotherapy	Radiotherapy delivered with sLITT in most cases; stabilization according to clinical need	Thoracic MESCC; functional status assessed in a combined-treatment context	NR	Radiographic and functional stability reported in the radiotherapy-integrated pathway	Not designed as a perioperative comparator	Complications and reinterventions reported by the source; definitions varied	Approximately 80% one-year freedom from local failure and 78% one-year overall survival; probable overlap with the institutional program series

**Table 2:** Detailed outcome summary across primary clinical reports ESCC: epidural spinal cord compression; EQ-5D: EuroQol five-dimension questionnaire; KPS: Karnofsky Performance Status; MESCC: metastatic epidural spinal cord compression; NR: not reported or not extractable; sLITT: spinal laser interstitial thermal therapy; VAS: visual analogue scale The ESCC classification, KPS, VAS, and EQ-5D are cited in the Methods section [12,19-21].

Study	Radiographic or ESCC outcome	Neurological or functional outcome	Pain or quality of life	Perioperative metrics	Complications and safety	Follow-up, local control, or survival
Tatsui et al. (2015) [9]	Median ESCC grade 2-1b; epidural thickness reduced	No permanent neurological morbidity; one transient sensory deficit	VAS and EQ-5D improved at 30 and 60 days	Median stay 2 days	One transient sensory deficit with spinal cord T2-weighted signal change; resolved	Median follow-up 4.7 months; imaging at approximately 2 months
Tatsui et al. (2016) [10]	Tumor size decreased in most patients	No permanent neurological injury reported	Clinical improvement reported; exact validated values NR	Short recovery described; exact metrics NR	No permanent neurological injury reported	Short-to-intermediate follow-up reported; exact value NR
Tatsui et al. (2016) [13]	Mean modified ESCC 4.5 ± 0.27 to 2.75 ± 0.37; p = 0.0044	KPS and clinical outcomes reported	NR	Median stay 5 days	NR in extracted dataset	Median imaging follow-up 2.1 months
Tatsui et al. (2017) [11]	Median ESCC grade 2-1b; median targeting radial error 0.7 mm	KPS and clinical outcomes reported	NR	Median stay 1 day	NR in extracted dataset	Median imaging follow-up 4.4 months
de Almeida Bastos et al. (2020) [14]	ESCC reduction 72.5% with sLITT versus 90% with open surgery	Standardized neurological outcome not uniformly reported	NR	Blood loss 117 ± 84 vs 1,331 ± 1,219 mL; stay 3.4 ± 4.3 vs 9.0 ± 5.9 days; time to radiosurgery 7.8 ± 11.8 vs 35.9 ± 32.7 days; time to systemic therapy 24.7 ± 21 vs 59 ± 80 days	Overall complications 5% vs 35%; source-defined major 2.5% vs 10%; source-defined minor 2.5% vs 25%	Follow-up not consistently extractable in review dataset
Bastos et al. (2021) [15]	Program-level decompression and local-control outcomes reported	Clinical outcomes reported at program level	NR	Approximately 85% discharged home within 48 hours	Overall complication rate 18%, mostly transient or procedural	Longer program follow-up; probable overlap with other reports
Tom et al. (2024) [16]	Thoracic MESCC outcomes reported with sLITT and radiotherapy	Functional stability reported in a radiotherapy-integrated context	NR	Not a primary perioperative comparison	Complications reported; definitions varied	Approximately 80% one-year freedom from local failure and 78% one-year overall survival

Population and Intervention Scope

Most reports focused on metastatic epidural spinal disease with radiographic ESCC, MESCC, or spinal canal compromise. Formal ESCC grade was reported in several studies but was not uniformly required across all reports. The evidence was therefore indirect for strictly defined symptomatic MSCC because some patients were selected according to radiographic epidural compression, spinal canal compromise, or neural-element-adjacent disease rather than a uniform clinical MSCC definition. The included pathways comprised standalone magnetic resonance-guided sLITT, hybrid sLITT with stabilization, sLITT used as a minimally invasive alternative to open separation surgery, program-level sLITT experience, and sLITT integrated with radiotherapy, as summarized in Table 3. The assessment of potential cohort overlap is provided in Appendix C.

**Table 3:** Intervention pathways and population scope ESCC: epidural spinal cord compression; MESCC: metastatic epidural spinal cord compression; sLITT: spinal laser interstitial thermal therapy

Pathway	Main reports	Population boundary	Outcome emphasis	Interpretation
Standalone magnetic resonance-guided sLITT	Tatsui et al. [9-11]	Definite ESCC or metastatic epidural/neural-element-adjacent disease in most reports	Radiographic decompression, neurological stability, pain, and quality of life in early series	Most directly relevant to technical feasibility of image-guided sLITT
Hybrid sLITT plus stabilization	Tatsui et al. [13]	ESCC with spinal instability	ESCC improvement, length of stay, and technical feasibility	Different from standalone sLITT because stabilization may influence recovery and outcomes
sLITT as an alternative to open separation surgery	de Almeida Bastos et al. [14]	Thoracic metastatic ESCC grade ≥1c	Perioperative comparison, ESCC reduction, and complications	Only direct matched comparison; observational and affected by treatment selection
Program-level sLITT experience	Bastos et al. [15]	ESCC from metastatic tumors	Safety, discharge, decompression, and program outcomes	Largest experience, but likely overlaps with earlier and later publications
sLITT integrated with radiotherapy	Tom et al. [16]	Thoracic MESCC	Local control, survival, and multidisciplinary treatment integration	Retrospective combined-treatment pathway; outcomes cannot be attributed to sLITT alone

Neurological and Radiographic Outcomes

Radiographic response was most often reported as ESCC improvement or reduction in epidural tumor thickness. Tatsui et al. reported a median ESCC improvement from grade 2 to grade 1b at approximately two months, together with reduced epidural tumor thickness [9]. A subsequent clinical series reported tumor-size reduction in most patients and no permanent neurological injury after magnetic resonance-guided sLITT followed by stereotactic radiotherapy [10]. The hybrid sLITT and stabilization series reported improvement in mean source-defined modified ESCC score from 4.5 ± 0.27 to 2.75 ± 0.37 at a median imaging follow-up of 2.1 months [13]. The technical accuracy study reported a median ESCC improvement from grade 2 to grade 1b and a median targeting radial error of 0.7 mm [11].

Neurological outcomes were generally reported as stability or absence of a new permanent neurological deficit. Standardized neurological scales and ambulatory outcomes were not reported consistently, which limited the evaluation of durable neurological recovery. The available evidence therefore supports short-term neurological preservation in selected patients more strongly than proven neurological improvement or long-term functional benefit.

Pain and Quality-of-Life Outcomes

Pain and quality-of-life reporting was sparse. The 2015 series used the VAS and the EQ-5D before treatment and at 30 and 60 days and reported improvement following sLITT [9]. Other studies did not consistently use validated pain or quality-of-life instruments, preventing meaningful quantitative comparison across reports. These outcomes were therefore considered supportive but very-low-certainty evidence.

Perioperative and Resource-Use Outcomes

Perioperative findings generally supported a minimally invasive recovery profile. Early reports described median hospital stays of approximately one to five days, depending on whether sLITT was performed alone or as part of a hybrid stabilization pathway [9,11,13]. In the matched observational comparison by de Almeida Bastos et al., sLITT was associated with lower estimated blood loss than open surgery (117 ± 84 mL versus 1,331 ± 1,219 mL), shorter length of stay (3.4 ± 4.3 days versus 9.0 ± 5.9 days), shorter time to stereotactic radiosurgery (7.8 ± 11.8 days versus 35.9 ± 32.7 days), and shorter time to systemic therapy (24.7 ± 21 days versus 59 ± 80 days) [14]. These results suggest potential perioperative advantages in selected patients, although residual confounding and treatment-selection bias remain important.

Safety and Complications

Complication reporting varied across studies. Tatsui et al. reported one transient sensory deficit associated with transient spinal cord signal change; the deficit resolved [9]. Early reports generally described no permanent neurological injuries, although definitions and follow-up intervals differed [9-11,13]. In the matched comparison, overall complications occurred in 5% of the sLITT group and 35% of the open-surgery group; source-defined major complications occurred in 2.5% and 10%, respectively, and source-defined minor complications occurred in 2.5% and 25%, respectively [14]. The 120-case institutional series reported an overall complication rate of 18%, with most events described as transient or procedural rather than durable neurological morbidity [15]. No pooled complication estimate was calculated because of probable cohort overlap and inconsistent definitions.

Local Control, Survival, and Integration With Radiotherapy

sLITT was frequently integrated with stereotactic radiosurgery, stereotactic body radiotherapy, or external beam radiotherapy. The 2024 cohort by Tom et al. evaluated sLITT with radiotherapy for thoracic MESCC and reported approximately 80% one-year freedom from local failure and 78% one-year overall survival [16]. These findings support the use of sLITT within a multidisciplinary pathway, but the retrospective design, combined treatment exposure, and likely overlap with the institutional program series prevent the attribution of oncological outcomes to sLITT alone.

Risk of Bias and Certainty of Evidence

The complete study-level critical appraisal results are presented in Table 4. Under the JBI checklist, all six non-comparative reports were judged to have clear eligibility criteria, reliable identification of the clinical condition, adequate demographic and clinical descriptions, clear reporting of the study site, and appropriate descriptive analysis. Consecutive and complete inclusion were unclear in five reports and were clearly reported only in the consecutive 120-case institutional series. Outcome and follow-up reporting was unclear in three early reports because clinical outcomes were secondary, incompletely standardized, or sparsely reported. For the matched non-randomized comparison, ROBINS-I judgments were serious for confounding and participant selection, low for intervention classification, and moderate for deviations from intended interventions, missing data, outcome measurement, and selection of the reported result. The overall ROBINS-I judgment was serious risk of bias.

**Table 4:** Complete risk-of-bias and critical appraisal results JBI items: Q1, clear inclusion criteria; Q2, standard and reliable measurement of the condition; Q3, valid methods for condition identification; Q4, consecutive inclusion; Q5, complete inclusion; Q6, clear demographic reporting; Q7, clear clinical reporting; Q8, clear outcome and follow-up reporting; Q9, clear reporting of the presenting site; Q10, appropriate statistical analysis. ROBINS-I domains: D1, confounding; D2, participant selection; D3, intervention classification; D4, deviations from intended interventions; D5, missing data; D6, outcome measurement; D7, selection of the reported result. Unclear indicates insufficient reporting in the source publication. Assessments followed ROBINS-I [22] and the JBI Case Series Checklist [23]; no numerical JBI score was calculated. JBI: Joanna Briggs Institute; ROBINS-I: Risk Of Bias In Non-randomized Studies of Interventions

Study	Appraisal tool	Complete assessment result	Overall interpretation
Tatsui et al. (2015) [9]	JBI Case Series Checklist	Q1 Yes; Q2 Yes; Q3 Yes; Q4 Unclear; Q5 Unclear; Q6 Yes; Q7 Yes; Q8 Yes; Q9 Yes; Q10 Yes	Included with limitations related mainly to unclear consecutive and complete inclusion and small sample size
Tatsui et al. (2016) [10]	JBI Case Series Checklist	Q1 Yes; Q2 Yes; Q3 Yes; Q4 Unclear; Q5 Unclear; Q6 Yes; Q7 Yes; Q8 Unclear; Q9 Yes; Q10 Yes	Included with limitations related to unclear recruitment completeness and incompletely standardized outcome reporting
Tatsui et al. (2016) [13]	JBI Case Series Checklist	Q1 Yes; Q2 Yes; Q3 Yes; Q4 Unclear; Q5 Unclear; Q6 Yes; Q7 Yes; Q8 Unclear; Q9 Yes; Q10 Yes	Included with limitations related to the small hybrid cohort, unclear recruitment completeness, and sparse outcome reporting
Tatsui et al. (2017) [11]	JBI Case Series Checklist	Q1 Yes; Q2 Yes; Q3 Yes; Q4 Unclear; Q5 Unclear; Q6 Yes; Q7 Yes; Q8 Unclear; Q9 Yes; Q10 Yes	Included with limitations related to unclear recruitment completeness and the technical rather than clinical outcome emphasis
Bastos et al. (2021) [15]	JBI Case Series Checklist	Q1 Yes; Q2 Yes; Q3 Yes; Q4 Yes; Q5 Yes; Q6 Yes; Q7 Yes; Q8 Yes; Q9 Yes; Q10 Yes	The most completely reported case series, although single-center selection effects and cohort overlap remained
Tom et al. (2024) [16]	JBI Case Series Checklist	Q1 Yes; Q2 Yes; Q3 Yes; Q4 Unclear; Q5 Unclear; Q6 Yes; Q7 Yes; Q8 Yes; Q9 Yes; Q10 Yes	Included with limitations related to retrospective selection, combined treatment, and uncertain cohort overlap
de Almeida Bastos et al. (2020) [14]	ROBINS-I	D1 Serious; D2 Serious; D3 Low; D4 Moderate; D5 Moderate; D6 Moderate; D7 Moderate	Serious overall risk of bias, driven primarily by residual confounding and non-random participant selection

Using GRADE, certainty was judged very low for radiographic decompression, neurological or functional stability, pain and quality of life, complications, and local control or survival. Certainty was judged low for perioperative recovery because one matched observational comparison provided objective comparative data, although serious residual confounding and selection bias remained. The outcome-level certainty judgments and reasons for downgrading are summarized in Table 5.

**Table 5:** GRADE certainty of evidence by outcome domain ESCC: epidural spinal cord compression; EQ-5D: EuroQol five-dimension questionnaire; GRADE: Grading of Recommendations Assessment, Development, and Evaluation; sLITT: spinal laser interstitial thermal therapy; VAS: visual analogue scale

Outcome domain	Evidence summary	Certainty	Main reasons for judgment
Radiographic decompression or ESCC response	Several small observational reports described ESCC improvement or reduced epidural tumor thickness	Very low	Serious risk of bias, probable cohort overlap, inconsistent definitions, short imaging follow-up, and imprecision
Neurological or functional stability	Most reports described stability or absence of permanent deterioration rather than standardized recovery	Very low	Non-comparative evidence, selected populations, inconsistent neurological scales, and imprecision
Pain and quality of life	Improvement was reported in one early series using VAS and EQ-5D; other studies rarely measured these outcomes	Very low	Sparse data, inconsistent instruments, serious risk of bias, and imprecision
Perioperative recovery	Brief hospital stay in series; one matched comparison reported lower blood loss, shorter stay, and faster transition to adjuvant therapy	Low	Objective outcomes and a comparative study increased confidence, but residual confounding, selection bias, and single-center evidence remained
Complications	Low major neurological morbidity in early reports; comparative study favored sLITT; 120-case series reported 18% overall complications	Very low	Different definitions, probable overlap, incomplete severity grading, and inconsistent follow-up
Local control and survival	Radiotherapy-integrated and program-level reports suggested encouraging one-year outcomes	Very low	Retrospective combined-treatment evidence, confounding by tumor biology and adjuvant therapy, no definitive comparator, and imprecision

Discussion

This systematic review and narrative synthesis evaluated image-guided sLITT for metastatic epidural spinal disease, including MSCC, MESCC, and related neural-element-adjacent metastatic disease. The available evidence suggests that sLITT can be performed in selected patients and may provide short-term radiographic decompression, neurological stability, brief hospitalization, and timely transition to radiotherapy or systemic treatment. However, confidence in these findings remains limited by serious or recurrent risk-of-bias concerns, single-institution reporting, probable cohort overlap, heterogeneous outcome definitions, and low or very low certainty of evidence.

The review differs from the prior systematic review by Cardia et al. [17] by extending the search through January 2026, including the 2024 cohort by Tom et al. [16], distinguishing primary clinical reports from secondary reviews, evaluating likely institutional cohort overlap, separating standalone, hybrid, comparative, and radiotherapy-integrated pathways, and avoiding pooling of potentially overlapping or clinically heterogeneous data. This approach is conservative but better reflects the limitations of the available evidence.

The most direct evidence comes from a small number of institutional series and one matched observational comparison. Early reports by Tatsui and colleagues established the technical feasibility of magnetic resonance-guided sLITT and suggested short-term ESCC improvement, pain improvement, and the preservation of neurological function in carefully selected patients [9-11]. The hybrid technical series extended the approach to patients requiring percutaneous stabilization [13]. The matched comparison by de Almeida Bastos et al. suggested lower blood loss, shorter hospitalization, fewer complications, and faster transition to adjuvant therapy than open surgery, although the ROBINS-I assessment indicates that selection bias and residual confounding limit causal interpretation [14].

The potential role of sLITT is best understood within a multidisciplinary framework. In selected patients, laser ablation may function as a minimally invasive separation strategy that reduces epidural tumor burden, facilitates stereotactic radiotherapy, and reduces delays associated with more extensive open procedures. sLITT should not be interpreted as a replacement for open decompression, stabilization, or radiotherapy. Rather, it may expand treatment options for patients who are medically frail, have focal disease suitable for a percutaneous approach, or require rapid return to systemic or radiation therapy.

Safety findings are encouraging but remain uncertain. Major permanent neurological complications were uncommon in the published reports, and the matched comparison favored sLITT for overall complications [14]. Nevertheless, inconsistent complication definitions, variable follow-up, selected patient populations, and possible repeated reporting from the same institutional experience reduce confidence. The 120-case series provides useful program-level experience, but probable overlap with earlier and later reports means that it cannot be combined with those studies to produce a cumulative complication rate [15].

The broader minimally invasive spine oncology literature supports the general principle that less invasive approaches may reduce blood loss, length of stay, and perioperative morbidity in selected patients [25-29]. This evidence is indirect for sLITT and does not establish comparative efficacy. It does, however, provide clinical context for interest in image-guided ablation among patients with advanced malignancy and limited physiological reserve.

Overall, the evidence is stronger for technical feasibility and short-term perioperative recovery than for durable neurological benefit, survival, or long-term local control. Oncological outcomes are influenced by tumor biology, radiation dose, systemic therapy, baseline ESCC grade, spinal stability, and patient selection. Future research should therefore use prospective multicenter registries or comparative studies, standardized ESCC reporting, predefined neurological and ambulatory endpoints, validated pain and quality-of-life measures, uniform complication grading, and transparent reporting of whether outcomes are patient-level, lesion-level, or procedure-level.

Limitations of the Study

This review has several limitations. The review was not prospectively registered, and no public protocol was available. Although registration was not required when the review was initiated, its absence limits verification of which methods and outcomes were prespecified. The inclusion criteria were intentionally broad to capture a small emerging evidence base, introducing indirectness because not all participants had uniform symptomatic MSCC. The interventions varied across standalone sLITT, hybrid sLITT with stabilization, comparative sLITT and open surgery, and sLITT integrated with radiotherapy. Several reports originated from the same institutional program and likely included overlapping patients. Outcome reporting was heterogeneous, follow-up was frequently short, and validated neurological, functional, pain, and quality-of-life instruments were not used consistently. Finally, no quantitative meta-analysis was performed, so the review cannot provide pooled estimates of treatment effect, complication rate, local control, or survival.

## Conclusions

Image-guided sLITT appears technically feasible for carefully selected patients with metastatic epidural spinal disease, including MSCC and radiographic MESCC. The available evidence most consistently supports short-term radiographic decompression, brief hospitalization, and potential perioperative recovery advantages. Evidence for durable neurological benefit, survival, and long-term local control remains uncertain because of observational design, serious or recurrent risk of bias, probable cohort overlap, short follow-up, and heterogeneous reporting. Prospective multicenter studies with standardized neurological, radiographic, safety, and patient-reported outcomes are required to define the role of sLITT within contemporary multidisciplinary spine oncology care.

## Appendices

Appendix A

**Table 6:** Complete database-specific search strategies and yields ESCC: epidural spinal cord compression; LITT: laser interstitial thermal therapy; MESCC: metastatic epidural spinal cord compression; MR: magnetic resonance; MRI: magnetic resonance imaging; sLITT: spinal laser interstitial thermal therapy The database searches identified 312 records in total; citation searching and other sources identified a further 18 records.

Database and platform	Search date	Coverage	Limits	Records identified	Complete search strategy
PubMed, National Library of Medicine	31 January 2026	Inception to 31 January 2026	No date or language restrictions	68	((spine[tiab] OR spinal[tiab] OR vertebral[tiab] OR epidural[tiab] OR "Spine"[Mesh]) AND (metastasis[tiab] OR metastases[tiab] OR metastatic[tiab] OR malignan*[tiab] OR tumor[tiab] OR tumour[tiab] OR neoplasm*[tiab]) AND ("spinal cord compression"[tiab] OR "epidural spinal cord compression"[tiab] OR ESCC[tiab] OR MESCC[tiab] OR "spinal canal compromise"[tiab] OR "thecal sac"[tiab] OR "cord compression"[tiab]) AND ("laser interstitial thermal therapy"[tiab] OR LITT[tiab] OR sLITT[tiab] OR "laser ablation"[tiab] OR "laser thermotherapy"[tiab] OR "thermal MRI"[tiab] OR "MR thermometry"[tiab]))
Embase, Elsevier	31 January 2026	Inception to 31 January 2026	No date or language restrictions	92	('spine'/exp OR spine:ti,ab OR spinal:ti,ab OR vertebral:ti,ab OR epidural:ti,ab) AND ('metastasis'/exp OR metastasis:ti,ab OR metastases:ti,ab OR metastatic:ti,ab OR malignan*:ti,ab OR tumor:ti,ab OR tumour:ti,ab OR neoplasm*:ti,ab) AND ('spinal cord compression'/exp OR 'spinal cord compression':ti,ab OR 'epidural spinal cord compression':ti,ab OR ESCC:ti,ab OR MESCC:ti,ab OR 'spinal canal compromise':ti,ab OR 'thecal sac':ti,ab OR 'cord compression':ti,ab) AND ('laser interstitial thermal therapy':ti,ab OR LITT:ti,ab OR sLITT:ti,ab OR 'laser ablation':ti,ab OR 'laser thermotherapy':ti,ab OR 'thermal MRI':ti,ab OR 'MR thermometry':ti,ab)
Scopus, Elsevier	31 January 2026	Inception to 31 January 2026	No date or language restrictions	86	TITLE-ABS-KEY((spine OR spinal OR vertebral OR epidural) AND (metastasis OR metastases OR metastatic OR malignan* OR tumor OR tumour OR neoplasm*) AND ("spinal cord compression" OR "epidural spinal cord compression" OR ESCC OR MESCC OR "spinal canal compromise" OR "thecal sac" OR "cord compression") AND ("laser interstitial thermal therapy" OR LITT OR sLITT OR "laser ablation" OR "laser thermotherapy" OR "thermal MRI" OR "MR thermometry"))
Web of Science Core Collection, Clarivate	31 January 2026	Inception to 31 January 2026	No date or language restrictions	66	TS=((spine OR spinal OR vertebral OR epidural) AND (metastasis OR metastases OR metastatic OR malignan* OR tumor OR tumour OR neoplasm*) AND ("spinal cord compression" OR "epidural spinal cord compression" OR ESCC OR MESCC OR "spinal canal compromise" OR "thecal sac" OR "cord compression") AND ("laser interstitial thermal therapy" OR LITT OR sLITT OR "laser ablation" OR "laser thermotherapy" OR "thermal MRI" OR "MR thermometry"))

Appendix B

**Table 7:** Full-text exclusion categories LITT: laser interstitial thermal therapy

Reason for exclusion	Number of reports
Wrong intervention or no laser component	7
Review article or narrative background source only	6
No extractable clinical outcomes	5
Intracranial-only LITT	4
Conference abstract only or insufficient data	3
Wrong population, benign disease, or no epidural/neural-element relevance	2
Overlapping report not retained as independent primary outcome evidence	1
Total excluded full-text reports	28

Appendix C

**Table 8:** Assessment of potential cohort overlap ESCC: epidural spinal cord compression; MESCC: metastatic epidural spinal cord compression; sLITT: spinal laser interstitial thermal therapy

Study	Institution or program	Recruitment or reporting context	Sample size	Overlap assessment	Use in synthesis
Tatsui et al. (2015) [9]	MD Anderson Cancer Center	Early feasibility experience; recruitment period reported in source	11	Likely overlaps with later program reports	Used for early feasibility, ESCC, pain, quality of life, and short-term safety; not added to cumulative totals
Tatsui et al. (2016) [10]	MD Anderson Cancer Center	Early clinical series of sLITT followed by stereotactic radiotherapy	19	Likely overlaps with early and later institutional reports	Used narratively for radiographic and neurological outcomes; not added to cumulative totals
Tatsui et al. (2016) [13]	MD Anderson Cancer Center	Hybrid sLITT plus percutaneous instrumentation series	8	Possible overlap with program experience, but clinically distinct hybrid pathway	Used for hybrid pathway description, ESCC outcome, and length of stay
Tatsui et al. (2017) [11]	MD Anderson Cancer Center	Technical accuracy study with clinical outcomes	13	Possible overlap with other institutional reports	Used for targeting accuracy and ESCC findings
de Almeida Bastos et al. (2020) [14]	MD Anderson Cancer Center	Matched comparison of sLITT and open surgery	80 total: 40 sLITT and 40 open surgery	Potential overlap with later program report	Used as the principal comparative evidence
Bastos et al. (2021) [15]	MD Anderson Cancer Center	Consecutive first 120 sLITT cases treated during 2013-2019	120 cases	Likely includes participants from earlier reports	Used as the largest program-level safety and experience report; not added cumulatively
Tom et al. (2024) [16]	MD Anderson Cancer Center	2013-2022 cohort of sLITT and radiotherapy for thoracic MESCC	129 patients; 144 vertebral segments	Likely overlaps with the program-level experience	Used for radiotherapy-integrated local control and survival outcomes; not added cumulatively
